# The Genetic Variant of SARS-CoV-2: would It Matter for Controlling the Devastating Pandemic?

**DOI:** 10.7150/ijbs.59137

**Published:** 2021-04-10

**Authors:** Shuxin Guo, Kefang Liu, Jun Zheng

**Affiliations:** 1 Faculty of Health Sciences, University of Macau, Macau SAR, China; 2 Chinese Academy of Sciences Key Laboratory of Pathogenic Microbiology and Immunology, Institute of Microbiology, Chinese Academy of Sciences, 100101 Beijing, China; 3 Institute of Translational Medicine, University of Macau, Macau SAR, China

**Keywords:** SARS-CoV-2, COVID-19, Genetic variant, D614G, VOC 202012/01, 501Y.V2

## Abstract

The pandemic of COVID-19, caused by severe acute respiratory syndrome coronavirus 2 (SARS-CoV-2), is far from being controlled despite the great effort that have been taken throughout the world. Herd immunity through vaccination is our major expectation to rein the virus. However, the emergence of widespread genetic variants could potentially undermine the vaccines. The evidence that some variants could evade immune responses elicited by vaccines and previous infection is growing. In this review, we summarized the current understanding on five notable genetic variants, i.e., D614G, Cluster 5, VOC 202012/01, 501Y.V2 and P.1, and discussed the potential impact of these variants on the virus transmission, pathogenesis and vaccine efficacy. We also highlight that mutations in the N-terminal domain of spike protein should be considered when evaluating the antibody neutralization abilities. Among these genetic variants, a concern of genetic variant 501Y.V2 to escape the protection by vaccines was raised. We therefore call for new vaccines targeting this variant to be developed.

## Introduction

Coronavirus Disease 2019 (COVID-19), caused by severe acute respiratory syndrome coronavirus 2 (SARS-CoV-2), has become an unprecedented challenge to globe since the 21^st^ century. This disease was initially emerged at the end of 2019 and the culprit was quickly identified as a novel coronavirus of SARS-COV-2 1-4. Being highly contagious and able to be transmitted by asymptomatic patients, SARS-COV-2 has quickly spread all over the world 1, 5, 6. As of 5 March 2021, there are in total 115 million people have been infected, resulting in over 2.5 million deaths globally. The devastating pandemic has badly disrupted the normal social activities and economic growth. Many cities around the world have been locked down and numerous people were quarantined; travel around the world was also restricted.

Numerous efforts have been tried out since the outbreak of COVID-19 to find effective therapeutic drugs and preventative vaccines that could help the return of our society to pre-pandemic normalcy. Among many drug candidates with high expectations, remdesivir have finally received authorization by the US Food and Drug Administration for emergency use as a treatment for COVID-19 7. However, the therapeutic efficacy is still under debate 8, 9. Till today, there is no drug potentially being a sovereign remedy for COVID-19. High expectation is now given to the vaccines developed against SARS-CoV-2. So far, a great many vaccine candidates are being developed, among which ten vaccines have completed their phase 3 trials and being approved for clinical use 10-12. However, virus evolution could undermine the COVID-19 vaccines. Despite the presence of a proofreading function in viral replication enables coronaviruses with lower rate of evolution compared to other RNA-viruses 13-15, genetic variants of SARS-CoV-2 have emerged 16 and several such variants have caused global concerns on the protective efficacy of currently developed vaccines 17-19. In this review, we will summarize the nature of five major genetic variants attracting public attention and discuss the potential impact of these variants as well as the mutations in N-terminal domain (NTD) of spike protein (S protein) on the control of the pandemic.

## A brief introduction on SARS-COV-2 and its S protein

SARS-CoV-2 is a single-strand, positive-sense RNA virus belonging to Beta-coronavirus genus 1, 5, 20. This genus also includes another two members known to infect human: severe acute respiratory syndrome coronavirus (SARS-CoV) and Middle East respiratory syndrome coronavirus (MERS-CoV), accountable for SARS (2002-2003) and MERS outbreaks respectively 21-24. The genome sequence identity of SARS-CoV-2 is up to 79% comparing with SARS-CoV and 50% with MERS-CoV 21. Compared to SARS-CoV and MERS-CoV, SARS-CoV-2 is more infectious but demonstrates lower case fatality rates 1.

There are at least 12 open reading frames (ORFs) in the genome of SARS-CoV-2, named as ORF1, Spike (S), ORF3, Envelope (E), Matrix (M), ORF7, ORF8, ORF9, ORF10b, Nucleocapsid (N), ORF13 and ORF14 21. ORF1 occupied approximate 67% in the whole encoding gene and encodes 15 non-structural proteins. Remaining ORFs encode 4 structural proteins (S protein, E protein, M protein and N protein) and accessory proteins 25.

**Fig 1:**
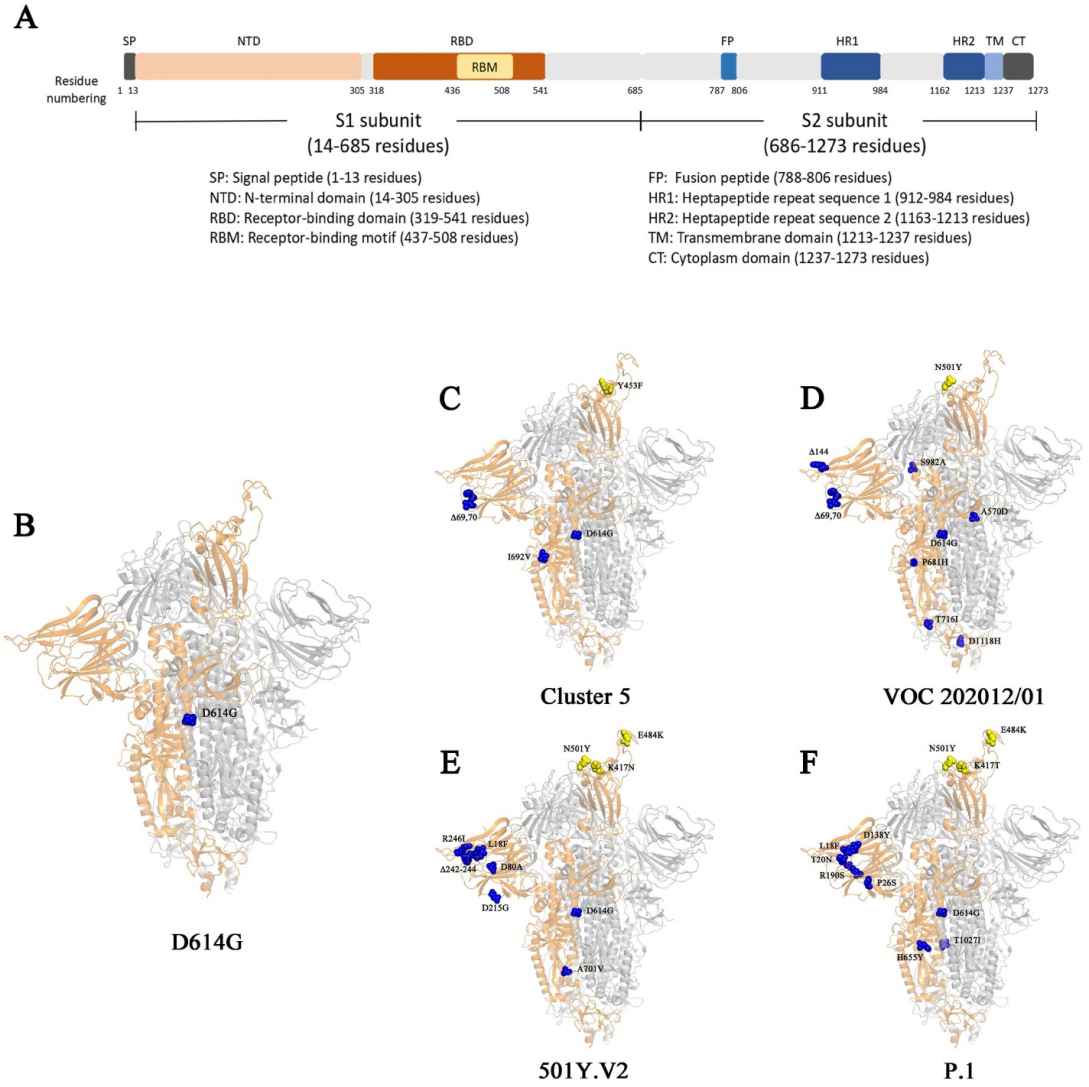
**The S protein and its mutations in different genetic variants**. (A) The schematic diagram of different domains in S protein. (B-F) The structural demonstration of mutations on S protein (PBD:6zgg) 97 in genetic variant D614G (B), Cluster 5 (C), VOC202012/01 (D), 501Y.V2 (E), and P.1 (F). The orange structure shows the monomer of spike protein; the yellow spheres represent the mutations on RBD; the blue spheres represent the mutations outside of RBD.

Surface glycoproteins are the most important elements for many viruses that are required for specific binding of cellular receptors, membrane fusion, and virus entry into the host cell 20. The epitopes of the surface glycoproteins are also recognized by neutralizing antibodies as part of an effective adaptive immune response. S protein in SARS-CoV-2 is the most important glycoprotein on the virion surface and is the main protein used as a target in COVID-19 vaccines 20. SARS-CoV-2 uses S protein to recognize and bind human angiotensin converting enzyme 2 (hACE2) on the surface of host cells 26, 27, and then enter host cells by endocytosis 28, 29. The S protein is a trimer. Each of its monomer has 1273 amino acids and is composed of two function subunits named S1 and S2 subunits, which is cleaved initially by Furin and then by the Transmembrane Serine Protease 2 (TMPRSS2) 27, 30-32. S1 contains a region that binds to receptors named receptor binding domain (RBD). S2 subunit is relatively conserved, helping membrane fusion during the virus infect cells (Fig. 1) 32, 33. Since outbreak of COVID-19, numerous genetic mutations were observed in SARS-CoV-2 isolates 34. The genetic variation in the SARS-CoV-2 surface glycoprotein is of paramount importance as the mutations are likely affect the vaccine effectiveness or immune escape of the virus.

## Important genetic variants of SARS-CoV-2

### D614G variant

The SARS-CoV-2 D614G variant, emerged at the end of January 2020, was first noticed in April 2020 in a preprint by Korber and colleagues, who warned of “D614G is increasing in frequency at an alarming rate” 35. The variant of D614G harbors a substitution of aspartic acid by a glycine at the position 614 of the virus spike glycoprotein, which helps virus particles to penetrate cells (Table 1) 35, 36.

During the infection, S protein of SARS-CoV-2 mediates the binding of the virus to ACE2 to gain cell entry 20, 32. The mutation of D614G attracted considerable attention as such mutation potentially could alter the receptor binding affinity, thus the virus infectivity and the immunogenicity 37. The importance of the D614G mutation was quickly seen by the analysis of the frequency of the ancestral strain D614 and D614G variant over time 35. The studies found that the location initially reporting the D614 viruses in the pandemic were often dominated by D614G virus subsequently 35, 38, 39. In addition, patients infected by the D614G variant demonstrated higher viral loads than that seen in the primary strain in the upper respiratory tract 35, 40.

It seems that variant D614G has enhanced abilities for infection and transmission 35, 39, 41-44. Initial experimental study on the D614G was performed using pseudovirus. Vesicular stomatitis virus and lentiviral particles incorporating the D614G variant were used to study the replication kinetics, and D614G demonstrated significantly higher pseudovirus titers in multiple cell types 35, 41. Subsequently, Planete and colleague engineered a D614G variant in the USA-WA1/2020 strain and used the resultant virus to infect human lung epithelial cells and the primary human airway tissue 42. They found that the mutation D614G enhanced viral replication through increasing the infectivity and stability of virions 42. In the Syrian golden hamster model, hamsters infected by D614G variant produced higher infectious titers in the nasal washes and the trachea, but not in the lungs, compared to those infected by D614 viruses 42. A competition assay comparing virus of D614G and D614 recovered the virus D614G /D614 ratio of 1.2 to 2.6, suggesting that D substitution by G at residue 614 of the S protein likely increases the virus fitness and transmission 42. These findings were echoed by another group, who used similar approach and concluded that D614G variant enhanced SARS-COV-2 infectivity, competitive fitness, and transmission in primary human cells and animal model 44. An enhanced entry of D614G was seen with pseudoviruses carrying D614G, which correlated with the observation of less S1 domain shedding and higher S protein incorporation into the virion 43. The enhanced transmissibility could also attribute to the increased stability of SARS-CoV-2 42. By comparing the decay of infectivity of D614 and D614G virus over time at 33℃, 37℃ and 42℃, the D614G variant retained higher infectivity at all temperatures than the D614 virus 42. However, it is of note that the analysis of a dataset of 46,723 SARS-CoV-2 genomes isolated from patients worldwide did not find any evidence associate with significantly increased viral transmission of variant D614G 45.

In contrast to the enhanced transmissibility, the mutation of D614G has little effect on the pathogenesis of SARS-CoV-2 in the hamster animal model 44. Consistently, patient infected by D614G did not show any altered disease severity 35, 40. To accurately recapitulate the effect of D614G variant on the virus transmissibility and clinical severity, Volz *et al*. examined D614G variant using more than 25,000 whole genome SARS-CoV-2 sequences from COG- UK dataset. They found that D614G was associated with higher viral load and younger age of patients 39. However, no increased COVID-19 mortality or clinical severity was found to be correlated with D614G 39. Interestingly, structural and functional analysis on D614G found that this mutation did not alter S protein synthesis, processing, or incorporation into SARS-CoV-2 particles, whereas the affinity of D614G to ACE2 was significantly reduced due to a faster dissociation rate 41. Conversely, another group showed an enhanced binding of D614G variant to hACE2 46.

Immunogenicity alteration is the most concern for the current efforts to control the pandemic by vaccination. Fortunately, the mutation in D614G variants seems not compromise the effectiveness of vaccines currently being developed against the ancestral SARS-COV-2. Kawaoka* et al*. used convalescent human serum and neutralizing antibodies to examine their blockage on live viruses of D614G variant and did not find any significant difference, consistent with the results from a separated study 37, 44. However, Plante *et al*. demonstrated that sera from hamsters infected with D614 virus have modestly higher neutralization titers against D614G variant than that against D614 virus 42. Consistently, Weissman and colleagues evaluated the neutralization of pseudoviruses bearing either D614 or D614G spike by sera from spike-immunized mice, non-human primates or convalescent sera from people infected with either form of the virus. They found that D614G was more susceptible to neutralization by all of the sera 47. Mechanism study on the enhanced vulnerability to neutralization by a negative stain electron microscopy revealed that the D614G spike has a higher percentage of the 1-RBD “up” conformation, which likely increases the epitope exposure to antibodies 47.Taken together, these results suggested that D614G mutation is unlikely reduce the ability of current vaccines to protect against COVID-19.

It is of note that D614G mutation has dominated the COVID-19 pandemic now (Table 2) 48 and all the variants mentioned below (Cluster 5, VOC 202012/01, 501Y.V2 and P.1.) carry the D614G mutation (Table 1).

### Cluster 5

In November 2020, the Danish public health authorities reported an outbreak of COVID-19 in North Jutland of Denmark that infected with mink-related virus variants (Table 2) 49. A unique variant stood out in 12 human cases (from 7 to 79 years) that was subsequently named as cluster 5, also called "ΔFVI" 49. In this variant, five mutations appeared on the S protein, including: Y453F, a H69/V70 deletion (ΔH69/ΔV70), I692V, S1147L and M1229I 49. The Y453F mutation locates in the RBD that directly contacts the host ACE2 at amino acid 34 27. The mutation I692V occurs seven amino acids downstream of the furin cleave site, whereas S1147L and M1229I locates on the S2 subunit (Table 1) 49. In addition, the simultaneous mutations of ΔH69/ΔV70 and Y453F were also found in several other variants closely related to cluster 5 with high frequency 49, 50.

Cluster 5 demonstrated an about 10-fold slower growth than SARS-CoV-2 wild type virus and several other variants isolated from patients at 24 h post inoculation of Vero E6 cells. However, a titer comparable to wild type but higher than other variants were seen at the time point from 24 h to 96 post inoculation 49. This slower growth of cluster 5 before 24 h post inoculation suggests a potential failure of diagnosis in the early infection stage.

The immunogenicity of variant cluster 5 has been assessed to address the concerns on a potentially reduced recognition of the virus by antibodies elicited by SARS-CoV-2 infection or vaccination. The results showed that the mutations in cluster 5 might moderately decrease sensitivity to neutralizing antibodies 49. In this study, the convalescent plasma with different neutralization titers was tested against cluster 5 variant. It was found that sera with different neutralization titer of wild type virus showed distinguishing neutralizing ability against Cluster 5. The neutralization activities of sera with high titers (n=2) were not affected, whereas those of plasma with low (n=4) and intermediate (n=3) titers were lost. An average of 3.58-fold decrease in neutralization activities of all 9 samples was observed 49. However, caution should be taken to interpret the results due to the small sample size.

Fortunately, following the strict measures by Denmark, the Cluster 5 seems already extinct (Table 2) 51. However, vigilance should be given for the transmission route between mink and human. In fact, a similar transmission between mink and human, which was caused by distinct genetic variant from cluster 5, has also been occurred in Netherlands 52.

### VOC 202012/01

The Variant of Concern (VOC) 202012/01 strain was originally derived from the SARS-CoV-2 20I/GR clade (also named as B.1.1.7 or 20I/501Y.V1), which emerged from South East England in September 2020 and has become the dominant strain in England in November/December 2020 (Table 2) 16, 53-55. It has expanded to 94 countries (Table 3) 56. The variant VOC 202012/01 bears 17 mutations, among which eight are located in the S protein: 6 substitutes and 2 deletions on S protein, 4 mutations on ORF1ab protein, 3 mutations on ORF8 protein and 2 mutations on N protein 57. Detailed information of mutations in VOC 202012/01 was shown in Table 1. The high number of novel mutations suggests that this variant might evolve from a single individual in a long-time infection, or from a geographic region with very poor sampling 58. Three mutations, namely N501Y, ΔH69/ΔV70 and P681H, locate in S protein. Residue N501 of S protein is one of the six critical amino acids interacting with ACE2 receptor 59. Mutant N501Y has demonstrated significant increase in the binding affinity to ACE2 60-62. ΔH69/ΔV70 has been circulating, separately and independently, or in other variant (such as in Cluster 5) for long 58. The mutation of ΔH69/ΔV70 seems unlikely to increase the risk of virus' escape from neutralizing antibodies. It was shown that ΔH69/ΔV70 either has a similar susceptibility as wild type to the neutralization by convalescent plasma 58, 63, or an enhanced sensitivity to the neutralization by the sera from the participants vaccinated by mRNA-1273 vaccine (Moderna) 64. However, ΔH69/ΔV70 could lead to the failure in the diagnosis by Thermopath TaqPath assay targeting S gene 55. The function of mutation of P681H is unclear but it locates near the furin-cleavage site, which is important for SARS-CoV-2 entry 65. In addition, the P681H mutation has appeared many times independently and has become dominant in the local epidemic in Hawaii 64.

VOC 202012/01 is highly transmissible compared to the parental strain of D614G. Using a mathematical model, it was found that VOC 202012/01 is 56% more transmissible than other preexisting variants of SARS-CoV-2 65, which might be the reason that had promoted the further national lockdown of UK. Another study predicted the *R_0_*of VOC 202012/01 is 1.75 times higher than the ancestral N501 strain, resulting in 75% more transmission 57. There is no any evidence demonstrating either the increased or decreased clinical severity of illness caused by VOC 2020/01 in the initial investigation 65. However, it is claimed recently that a possible increased death rate might associate with VOC 202012/01 66. The detailed information on this study is waiting to be disclosed 66.

It seems that variant VOC 202012/01 does not compromise the neutralization by antibodies elicited by previous infection or vaccination 64, 67-72. Mutation in N501 was previously shown to have modest effects on binding by some monoclonal antibodies but not by convalescent sera 67, 68, 73. Recently, Xie *et al* generated isogenic N501 and N501Y SARS-CoV-2 strains, and examined sera from participants vaccinated by mRNA-based COVID-19 vaccine BNT162b2 (Pfizer-BioNTech). They showed that the sera had equivalent neutralizing titers to the N501 and N501Y viruses 72. A similar study was conducted to examine the neutralization of SARS-CoV-2 pseudoviruses bearing either the Wuhan reference strain or the VOC 202012/01 by sera from participants vaccinated by BNT162b2. They found that the immune sera had equivalent neutralizing titer to both variants (Table 3) 69. Similarly, with a lentivirus-based pseudovirus assay, sera from recipients of either mRNA-1273 (Moderna) or protein nanoparticle NVX-CoV2373 (Novavax) vaccine were still able to neutralize VOC 202012/01, albeit at moderately reduced levels (~2 fold) (Table 3) 64, 70. Consistently, in a phase 3 trial, the vaccine of NVX-CoV2373 demonstrated 85.6% protective efficacy against VOC 202012/01 (Table 3) 71.

### 501Y.V2 and P.1

501Y.V2 (also called B.1.351 or 20H/501Y.V2) is another highly transmissible SARS-CoV-2 variant. It emerged from the first wave of the South African COVID-19 epidemic in the Eastern Cape province in early 2020 (Table 2) 34. However, it spread so quickly that it had become the predominant virus lineage in the Eastern and Western Cape province by the end of November 2020 34. This variant has been detected in 48 countries worldwide by March 2021 74.

The 501Y.V2 variant is characterized by carrying nine mutations in S protein (L18F, D80A, D215G, R246I, Δ242-244, K417N, E484K, N501Y, A701V) (Table 1) 75, three of which (K417N, E484K and N501Y) locate in the RBD of the S protein 34. The mutations of both N501Y and E484K locate in the receptor binding motif (RBM) in the RBD (Fig. 1) 32. N501Y is also presented in the VOC 202012/01 variant 53. These two mutations could profoundly affect the binding of the variant to host. Indeed, N501Y has been shown to increase the affinity to hACE2 receptor 60-62. Different from N501Y and E484K, the mutation of K417 locates outside the RBM. It forms a salt-bridge interaction with N30 of ACE2 76. SARS-CoV-2 RBD with a replacement of this residue by a valine failed to participate in ACE2 binding 76. However, the substitution by an asparagine showed an increase binding to ACE2 77. Consistently, preliminary results indicate that 501Y. V2 variant may also have an increased transmissibility 78. However, it is not confirmed yet whether the disease severity caused by 501Y. V2 variant is also changed 78.

Nevertheless, the extensive mutations occur in the RBD of 501Y.V2 attracted serious concerns on the potential escape from the antibodies against parental SARS-CoV-2. Indeed, a single mutation in reside 484 was previously shown to significantly reduce the neutralization by several monoclonal antibodies and sera 73, 79. Wibmer *et al*., recently used three classes of therapeutically relevant monoclonal antibodies to examine their neutralizations on 501Y.V2 75. Both class 1 and class 2 antibodies target site 1 epitopes that overlap with the ACE2 receptor binding site 80, 81. Class 1 antibodies are accessible to the RBD “up” conformation while class 2 antibodies can bind both “up” and “down” conformation of S protein. Class 3 antibodies target other regions that are outside the ACE2-binding site 81. The results showed that 501Y.V2 completely escaped from all these three classes of monoclonal antibodies 75. Furthermore, when plasma from individuals previously infected with SARS-CoV-2 was used, 501Y.V2 demonstrated significantly resistance 75. Similarly, Cele *et al*. examined the neutralization of convalescent plasma from patients infected by SARS-CoV-2 carrying D614G mutation but no mutation in RBD or NTD (Fig. 1). They found that the neutralization ability of sera to 501Y.V2 was significantly reduced 82. The conclusion of 501Y.V2 resistance to multiple classes of SARS-CoV-2 directed monoclonal antibodies and plasma was also reinforced by other groups 75.

Plasma collected from individuals vaccinated by Moderna and Pfizer-BioNTech vaccines were also examined against variants bearing mutations observed in 501Y.V2. The results showed that the neutralization activities of plasma from vaccinated individual to the pseudoviruses expressing N501Y, E484K or K417N:E484K:N510Y were significantly reduced 83. A preliminary study observed a two third weaker of the neutralizing activity by the BNT162b2-elicited serum against the pseudovirus that bears all mutations in S protein observed in 501Y.V2 84. However, the neutralization by sera from participants vaccinated with BNT162b2 against N501Y, Δ69/Δ70+ N501Y + D614G and E484K+ N501Y+D614G pseudoviruses only demonstrated small effects of these mutations on neutralization 85. Furthermore, Moderna has announced that its COVID-19 vaccine retained the neutralizing activity against 501Y.V2 and VOC 202012/01 despite that a six-fold decrease in the neutralizing efficacy was indeed observed (Table 3) 70. Similarly, the inactivated vaccine BBIBP-CorV and recombinant dimeric RBD vaccine ZF2001 were shown to largely preserve the neutralizing titers against 501Y.V2, with a slight reduction comparing to original strain or D614G strain (Table 3) 86. The preliminary data published on Novavax website also indicated that their protein-based COVID-19 vaccine candidate NVX-CoV2373 achieved 60% efficacy against 501Y.V2 in the Phase 2b clinical trial (Table 3) 71. However, a detailed data is still lacking. Nevertheless, Moderna has announced to prepare an emerging variant booster candidate (mRNA-1237.351) against the 501Y.V2 variant 70.

A similar variant to 501Y.V2 was identified from Brazil, which was named as variant P.1 (descendent of B.1.1.28), 20J/501Y.V3 or VOC202101/02 (Table 2) 16, 87, 88. It has been detected in 25 countries (Table 2) 89. P.1 has three mutations in the RBD of S proteins: K417T, E484K and N501Y (Table 1). The variant P.1 and 501Y.V2 seems developed independently 87. There is no any evidence of changes in transmissibility, severity, immunity, vaccination and diagnostic 78. However, genetic variant P.1 and 501Y.V2 are considered to bear similar functional characteristics because the mutations in the RBD of S protein occur at the same sites. Therefore, vaccine efficacy might also be compromised against P.1 if they failed on 501Y.V2.

### Variants with mutations in N-terminal domain of S protein

Neutralizing antibodies against SARS-CoV-2 mainly target RBD of the S protein which binds to hACE2 80. hACE2 is the well-known receptor for SARS-CoV-2. However, the expression level of hACE2 is extremely low in many human tissues, such as in the respiratory tract. It was shown that cells with low expression of ACE2 or without ACE2 expression could be infected by SARS-CoV-2 90. These observations suggest that alternative receptor(s) for SARS-CoV-2 binding might exist. Through screening the lung cDNA library, Soh *et al*. found two receptors (L-SIGN and DC-SIGN) that specifically bind NTD 91. Additionally, a new potent receptor of the tyrosine-protein kinase receptor UFO (AXL) was identified by another group to bind NTD 92. It was shown that overexpression of AXL in HEK293T cells promoted SARS-CoV-2 entry in a similar efficiency as those overexpressing ACE2. In contrast, knocking out AXL significantly reduces SARS-CoV-2 infection in pulmonary cells and lung epithelial cells 92. Importantly, AXL expresses in nearly all human organs, and a correlation of virus infection titers and AXL expression level in patients' samples was observed 92.

The finding of NTD as a binding site for human receptor AXL indicates its clinical significance. Indeed, antibodies targeting NTD can have the neutralizing ability 93, 94. Another evidence of the importance of NTD is the mass mutation observed from the genome sequences of SARS-CoV-2 isolates. Through analyzed 146,795 SARS-CoV-2 genome sequences, McCarthy *et al*. found that the deletions frequently occurred at four sites that defined antigenic site in the NTD of the S protein 95. Similar observation was reported by McCallum and colleagues, who also identified a vulnerability site containing three regions that can be easily mutated to escape the neutralizing stress in NTD 96.

Except D614G, all the critical variants we reviewed here contain mutations in NTD: for example, L18F in variant 501.V2 and P.1, T20N and P26N in variant P.1, ΔH69/ΔV70 in Cluster 5 and VOC 202012/01, Y144 deletion in VOC 202012/01, 242-244 deletion and R246I in VOC 202012/01. The detail mutations in different variants can be seen in Table 1. Therefore, consideration should be given to the mutations in NTD when we evaluate the antibody neutralization abilities.

## Conclusive remarks

SARS-CoV-2 is continuing to threaten human lives one year after the outbreak. Despite a great many vaccines have been developed or being under development, the emergence and quick spread of genetic variants with high transmission ability suggest that the current controlling measures may be invaded (Table 3). Furthermore, the fast-spreading of the SARS-CoV-2 variants may evade immune responses of recovered patients and undermine the vaccines being approved or under development. All countries should collaborate and work together to prevent the spreading of such variants. Research should be conducted to investigate the impact of these variants on vaccination, and new vaccines targeting the important new variant (such as 501Y.V2) should at least be technically prepared immediately.

## Figures and Tables

**Table 1:** Summary of mutations on S proteins in the five SARS-CoV-2 variants

Amino acid position in S protein	Wuhan-Hu-1	D614G	Cluster 5	VOC 202012/01	501Y.V2	P.1	Note
	Residues in S protein						
18	L	*	*	*	F	F	NTD
20	T	*	*	*	*	N	
26	P	*	*	*	*	S	
69,70	H, V	*	Delete	Delete	*	*	
80	D	*	*	*	A	*	
138	D	*	*	*	*	Y	
144	Y	*	*	Delete	*	*	
190	R	*	*	*	*	S	
215	D	*	*	*	G	*	
242-244	L, A, L	*	*	*	Delete	*	
246	R	*	*	*	I	*	
417	K	*	*	*	N	T	RBD
453	Y	*	F	*	*	*	
484	E	*	*	*	K	K	
501	N	*	*	Y	Y	Y	
570	A	*	*	D	*	*	
614	D	G	G	G	G	G	
655	H	*	*	*	*	Y	
681	P	*	*	H	*	*	
692	I	*	V	*	*	*	
701	A	*	*	*	V	*	
716	T	*	*	I	*	*	
982	S	*	*	A	*	*	Heptad repeat 1
1027	T	*	*	*	*	I	
1118	D	*	*	H	*	*	
1229	M	*	I	*	*	*	Transmembrane domain

* indicates the identical residues in the SARS-CoV-2 with the reference strain of Wuhan-Hu-1

**Table 2:** The summary of five notable genetic variants

Genetic Variant	Alternative name	Country and time initially isolated*	Spreading range	Note
D614G	-	Germany Feb 2020	Global 35	Increased transmission 42, 44; increased infectivity 42, 44
Cluster 5	-	Denmark Aug 2020	No	Transmission between human and mink 49; likely extinct 51
VOC 202012/01	B.1.1.7 20I/501Y.V1	England Sep 2020	94 countries reported 56	Increased transmission 57
501Y.V2	B.1.351 20H/501Y.V2	South Africa Oct 2020	48 countries reported 74	Possible immune escape 75
P.1	B.1.1.28.1 20J/501Y.V3 VOC202101/02	Brazil Dec 2020	25 countries reported 89	Unknown

* data were retrieved from GISAID

**Table 3:** The activities of different vaccines on two genetic variants

Vaccines	Type of vaccine	Supplier	Activities on genetic variants	
			VOC 202012/01	501Y.V2
BNT162b2	mRNA	Pfizer-BioNTech	Equivalent to wild type strain*^69^	Two - third reduction*^84^
mRNA-1273	mRNA	Moderna	2-fold reduction compared to D614G strain*^64^	6-fold reduction*^70^
NVX-CoV2373	Protein nanoparticle	Novavax	85.6% efficacy in Phase 3 clinical trial 71; 2-fold reduction compared to D614G strain*^64^	60% efficacy in Phase 2b clinical trial^71^
BBIBP	Inactivated	Sinopharm	No data	1.6-fold reduction compared to D164G strain*^86^
ZF2001	Recombinant dimeric RBD	Anhui Zhifei Longcom	No data	1.6-fold reduction compared to D164G strain*^86^

* refers to the neutralization activities of sera from participants vaccinated by the respective vaccines
